# The outcome and prognostic factors of 248 elderly patients with acute myeloid leukemia treated with standard-dose or low-intensity induction therapy

**DOI:** 10.1097/MD.0000000000004182

**Published:** 2016-07-29

**Authors:** Yi Chen, Ting Yang, Xiaoyun Zheng, Xiaozhu Yang, Zhihong Zheng, Jing Zheng, Tingbo Liu, Jianda Hu

**Affiliations:** Fujian Provincial Key Laboratory of Hematology, Fujian Institute of Hematology, Fujian Medical University Union Hospital, Fuzhou, Fujian, P. R. China.

**Keywords:** acute myeloid leukemia, elderly patients, induction therapy, low-intensity, standard-dose

## Abstract

The prognosis of elderly patients with acute myeloid leukemia (AML) is poor, and the recommendation of standard-dose or low-intensity induction regimen for these patients remains controversial. We retrospectively analyzed treatment outcome and prognostic factors of elderly AML patients who had received either standard-dose or low-intensity induction regimens.

Two hundred forty-eight elderly AML patients with good Eastern Cooperative Oncology Group performance status (ECOG PS ≤ 2) received one of three regimens for induction in this study: standard-dose cytarabine plus idarubicin (IA; n = 144) or daunorubicin (DA; n = 42); low-intensity cytarabine, aclarubicin, and granulocyte colony-stimulating factor (G-CSF) (CAG; n = 62).

After first induction treatment cycle, the overall complete remission (CR) rate was 42.7%. Patients in IA group had a higher CR rate than in DA or CAG group (49.3%, 35.7%, and 32.3%, respectively; *P* = 0.046). The 1-year, 3-year, and 5-year overall survival (OS) rates were 42.2%, 18.9%, and 13.5% for these 248 patients, with median survival of 9.2 months. Long-term survival of IA group was better than DA or CAG group. The 1-year, 3-year, and 5-year OS rates of IA group were 45.9%, 23.5%, and 19.4%, respectively, as compared to 39.8%, 8.3%, and estimated 2.4% in DA group, and 34.9%, 15.9%, and 6.3% in CAG group, respectively. Early induction mortality and 2-year relapse rates showed no difference among 3 groups. Univariate analysis and multivariate analysis identified lactic dehydrogenase (LDH) more than two times of upper normal limit at diagnosis and nonremission after first induction cycle as adverse prognostic factors for OS. A simple and valid scoring model was constructed for risk stratification and prediction of long-term survival of elderly AML patients.

Standard-dose IA regimen could improve the prognosis of elderly AML patients with good performance status compared with standard-dose DA or low-intensity CAG regimen. All prognostic factors and risk assessment should be considered to ensure that each patient receives the suitable individualized treatment.

## 1 Introduction

Acute myeloid leukemia (AML) is a disease of older adults, with a median age of 65 to 70 years. The majority of the approximately 14,500 individuals diagnosed with AML each year in the US are over the age of 60, and a third are over the age of 75.^[1]^ The treatment outcome of AML appears to be worse with increasing age, due to such adverse factors as poor performance status, comorbidity, higher frequency of unfavorable cytogenetic findings (monosomy 7, del (5q), complex karyotypes), frequent involvement of a more immature leukemic precursor clone, multidrug resistance mediated by multidrug resistance protein 1(MDR1)/P-glycoprotein, and the presence of antecedent hematopoietic disorders.^[2–4]^ Because of these complexities, treatment of elderly patients with AML remains highly challenging and controversial. The 5-year overall survival (OS) rate of adults ≥ 60 years old with AML is currently less than 20%.^[5–7]^

At present, standard-dose induction chemotherapy using cytarabine plus idarubicin (IA regimen) or daunorubicin (DA regimen) is generally considered to be the most effective upfront AML induction therapy.^[8]^ However, a number of elderly patients cannot tolerate this induction chemotherapy protocol because of poor performance status and other complications. Therefore, low-intensity chemotherapy, including a CAG regimen, which combines low-dose cytarabine, aclarubicin, and granulocyte colony-stimulating factor (G-CSF), was used for those patients who could not tolerate standard-dose chemotherapy. The safety and efficacy of the CAG regimen has been previously confirmed in some studies at several medical centers.^[9,10]^

Risk assessment was highlighted to screen the eligible patients for chemotherapy in National Comprehensive Cancer Network (NCCN) or European Society for Medical Oncology (ESMO) guidelines for older AML patients.^[11,12]^ Chemotherapy protocols for elderly AML include standard-dose intensive chemotherapy, low-intensity therapy, and palliative treatment, and the recommendation of standard-dose or low-intensity induction regimen for elderly AML patients remains controversial. Thus, we retrospectively compared the outcome and explored the prognostic factors of elderly patients with AML treated with standard-dose of IA or DA, or low-intensity of CAG induction chemotherapy regimens.

## 2 Patients and methods

### 2.1 Patients and inclusion criteria

Between January 2003 and April 2015, 248 elderly patients (≥ 60 years old) with newly diagnosed AML (except acute promyelocytic leukemia (APL) with t(15;17) (q22;q12); PML-RARA, subtypes) who were treated at our center were enrolled in this retrospective study. The patients included 137 men and 111 women, 226 with primary AML, 22 with secondary AML. The patients’ age ranged from 60 to 87 years old, with 162 cases between 60 and 69 years old, 74 cases between 70 and 79 years old, and 12 cases older than 80 years. All patients were unrelated ethnic Han Chinese. The diagnosis of AML was made according to French-American-British Cooperative Group (FAB) classification combined with the 2008 revision of the World Health Organization (WHO) classification of myeloid neoplasms and acute leukemia.^[13,14]^

The main inclusion criteria were followed, including age ≥ 60 years, Eastern Cooperative Oncology Group performance status (ECOG PS) score ≤ 2, and received IA, DA or CAG regimen for induction therapy. Patients with severe complications of heart, liver, kidney, or other important organ before induction therapy were excluded in this study. However, once the patients met the main inclusion criteria and were treated with these regimens, they were included in analysis, even if they developed above-mentioned severe complications.

This study was approved by Ethics Committee of Fujian Medical University Union Hospital.

### 2.2 Treatment

In each case, a standard-dose or low-intensity induction regimen was chosen by patient's preference. One hundred forty-four patients received an IA regimen (idarubicin 8 or 10 mg/m^2^ qd d1–3; cytarabine 100 mg/m^2^ q12 h d1-7). Forty-two patients received a DA regimen (daunorubicin 45 or 60 mg/m^2^ qd d1-3; cytarabine 100 mg/m^2^ q12 h d1-7). In IA or DA regimen, cytarabine could be reduced to 5 or 6 days in patients with serve neutropenia and infection during induction phase. Sixty-two patients received a CAG regimen (cytarabine 10 mg/m^2^ q12 h d1-14; aclarubicin 20 mg qd d1-4; G-CSF 300 ug/d d0-14, or until hematopoietic function of bone marrow recovered.).

After the patients achieved complete remission (CR) with induction therapy, most of them received 4 to 6 cycles of high-dose cytarabine (2 g/m^2^ q12 h d1-3, or 3 g/m^2^ q12 h d1-2) for consolidation. To date, only one patient received allogeneic hematopoietic stem cell transplantation (Allo-HSCT) after CR. When patients failed to respond to any of these 3 regimens, they would receive MA (mitoxantrone, cytarabine), FLAG (fludarabine, cytarabine, and G-CSF), or decitabine-based combination therapies as salvage therapies.

### 2.3 Response criteria and endpoint

Complete remission was defined by the presence of normal cellular bone marrow (BM) with fewer than 5% blasts along with neutrophil count ≥ 1.5 × 10^9^/L, platelet count ≥ 100 × 10^9^/L in peripheral blood (PB), and transfusion independent status.^[15]^ Relapse was defined as the reappearance of more than 5% leukemic blasts in the bone marrow or peripheral blood, or the presence of blast infiltration in extramedullary organs such as the central nervous system or other organ systems. Overall survival (OS) was defined as the period from the time of first diagnosis to death or censored on the last follow-up date if the patient was still alive.

### 2.4 Statistical analysis

Qualitative parameters were evaluated by χ^2^ test, and quantitative parameters were evaluated by *t* test. The OS was calculated by Kaplan–Meier method, and statistical significance was analyzed by log-rank test. Univariate and multivariate Cox proportional hazard models were used for exploring significantly prognostic clinical variables. All statistical analyses were performed using the Statistical Program for Social Sciences (SPSS) 21.0 (IBM Corp., Armonk, NY, USA). In all above statistical analysis, *P* < 0.05 was considered statistically significant.

## 3 Results

### 3.1 Outcome of treatment

**Figure 1:**
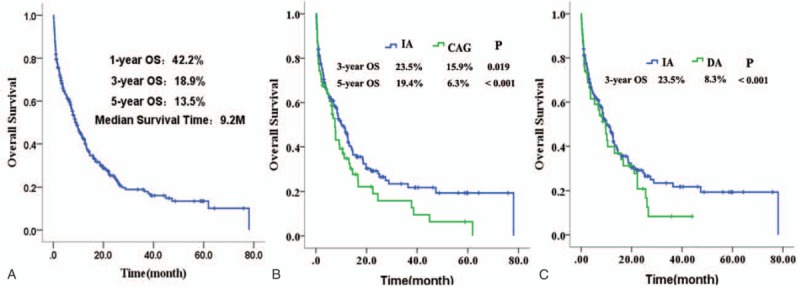
The overall survival (OS) of 248 patients. (A) All of 248 patients. (B) Patients treated with IA or CAG regimen. (C) Patients treated with IA or DA regimen. CAG = cytarabine, aclarubicin, and granulocyte colony-stimulating factor, DA = daunorubicin plus cytarabine, IA = idarubicin plus cytarabine.

The clinical baseline characteristics of 3 groups are listed in Table 1. White blood cell (WBC) count of IA group was higher than CAG group, and the CAG group was older than IA group. Bone marrow blast and lactic dehydrogenase (LDH) were not significantly different between IA and DA group, but both were higher in IA or DA than in CAG group. For all of these 248 patients, the median follow-up time was 27.1 months. After the first induction treatment cycle, the total CR rate was 42.7%. IA group had a higher CR rate than DA or CAG group (IA, 49.3%; DA, 35.7%; CAG, 32.3%; *P* = 0.046). The median OS for all 248 patients was 9.2 months, and the 1-year, 3-year, and 5-year OS rates were 42.2%, 18.9%, and 13.5%, respectively (Fig. 1A). The median OS for patients receiving IA, DA, and CAG induction therapy were 10.3 months, 9.7months, and 7.5 months, respectively(*P* = 0.127). The long-term survival of IA regimen was better than CAG or DA regimen. One-year, 3-year, and 5-year OS rates in IA group were 45.9%, 23.5%, and 19.4%, while they were 39.8%, 8.3%, and estimated 2.4% in DA group, and 34.9%, 15.9%, and 6.3% in CAG group, respectively (Fig. 1B and C). The early mortality of induction chemotherapy in the IA, DA, and CAG groups showed no difference (16.0% for IA, 16.7% for DA, and 21.0% for CAG group, *P* = 0.680). The 2-year relapse rates were 59.2%, 46.7%, and 45.0% in the IA, DA, and CAG groups, respectively (*P* = 0.423), with median time to relapse at 8.8 months, 13.7 months, and 11.5 months for the IA, DA, and CAG regimens, respectively (Table 2).

**Table 1:**
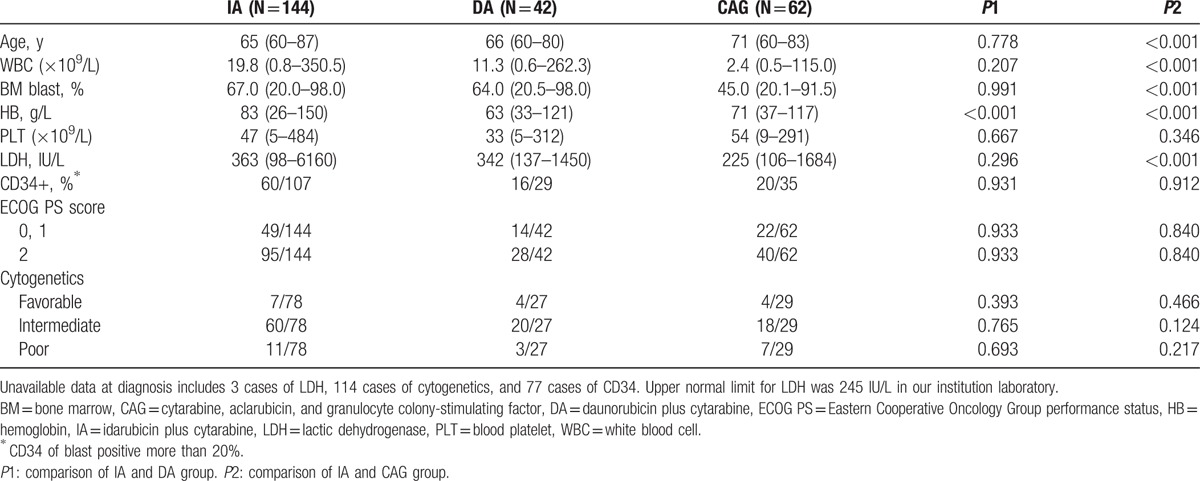
Patient baseline characteristics.

**Table 2:**
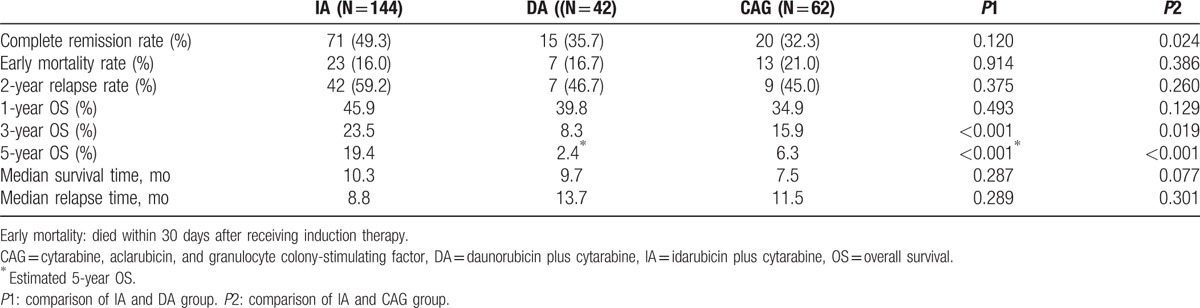
Treatment outcome of IA, DA, or CAG regimen.

### 3.2 Prognostic factors for OS

**Figure 2:**
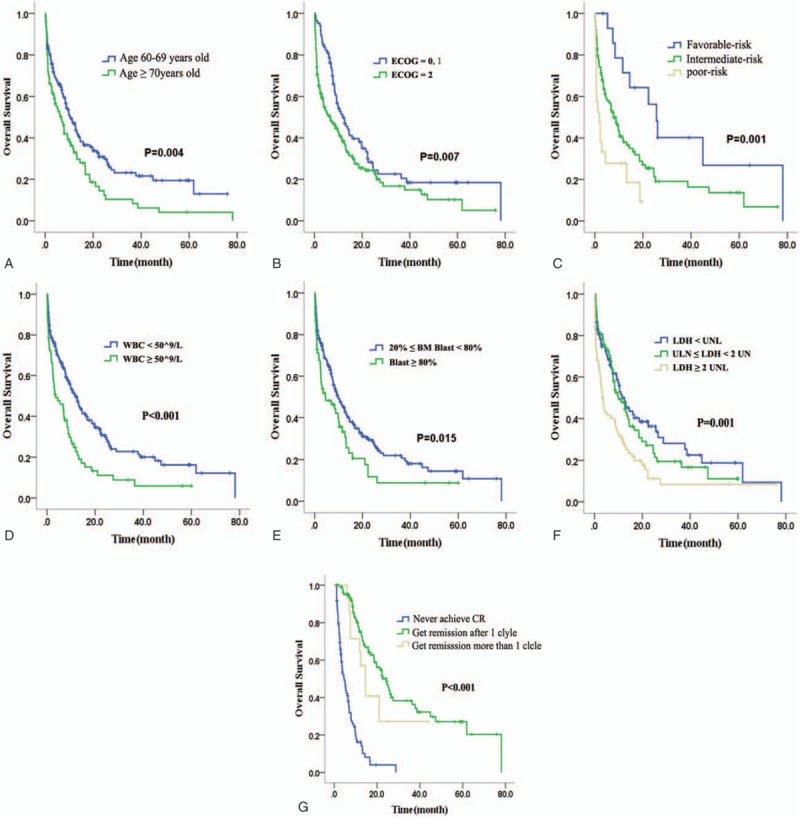
The overall survival (OS) of patients with different prognostic factors. (A) Age. (B) Eastern Cooperative Oncology Group (ECOG) score. (C) Cytogenetics. (D) White blood cell (WBC) count. (E) Bone marrow (BM) blast. (F) Lactic dehydrogenase (LDH). (G) Response to first induction cycle.

To identify the clinical prognostic factors of elderly AML patients, we performed survival analysis for these 248 elderly AML patients (Table 3). Univariate analysis identified 7 adverse prognostic factors of OS for elderly AML patients, including older age, poor ECOG PS, unfavorable cytogenetics, WBC ≥ 50 × 10^9^/L, percentage of BM blast ≥ 80%, higher LDH, and nonremission after first induction cycle (Fig. 2A–G). Previous hematologic diseases, hemoglobin, platelet count, and expression of CD34 at diagnosis had no impact on OS in these elderly AML patients.

**Table 3:**
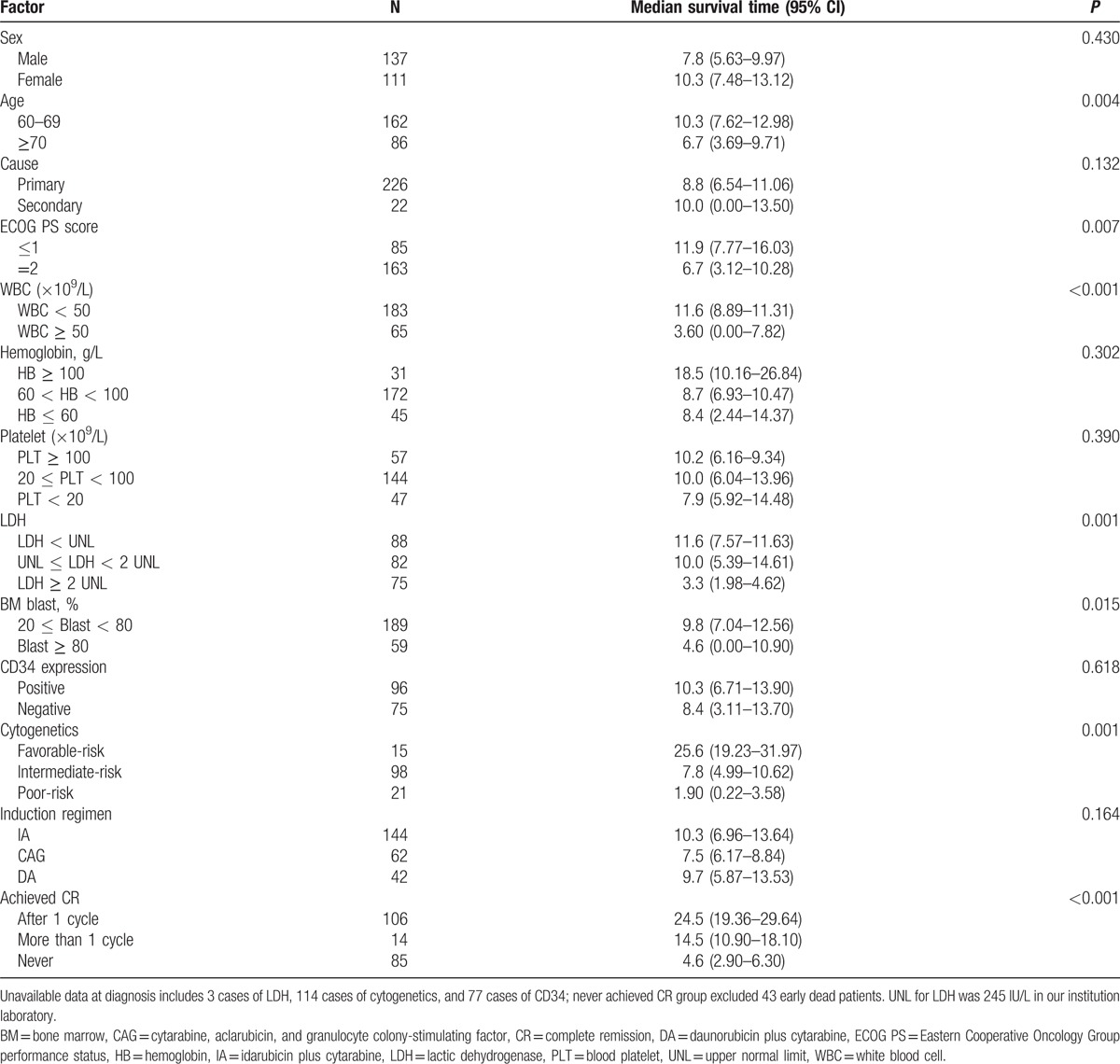
Univariate analysis of prognostic factors.

In multivariate analysis, we constructed a Cox proportional hazard model to evaluate the prognostic significance of the following parameters: age ≥ 70 years, ECOG PS score = 2, poor-risk cytogenetics, nonremission after first induction cycle, WBC count ≥ 50 × 10^9^/L, LDH ≥ 2 times upper normal limit (UNL), and percentage of BM blasts ≥ 80% (Table 4). Our data analysis defined 2 significantly independent prognostic parameters of OS, which were nonremission after first induction cycle (hazard ratio (HR) = 6.141, 95% CI: 3.585–10.52, *P* < 0.001) and LDH ≥ 2 times UNL (HR = 1.001, 95% CI: 1.000–1.001, *P* < 0.001).

**Table 4:**
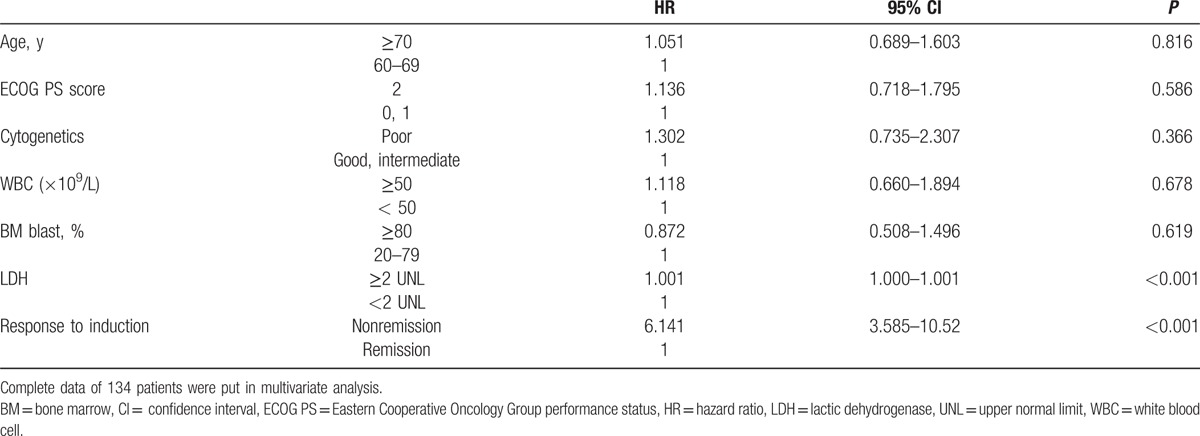
Multivariate analysis of prognostic factors.

**Figure 3:**
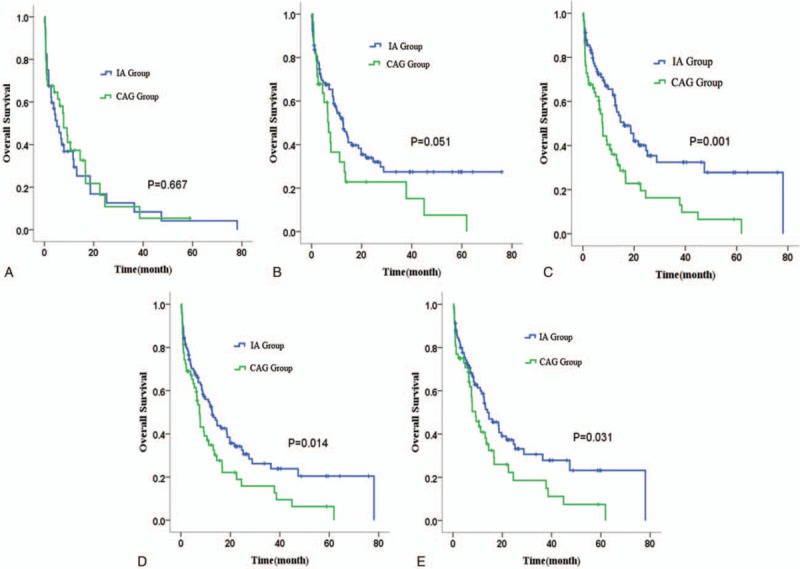
The overall survival (OS) of patients with different prognostic factors treated with IA or CAG regimen. (A) Age ≥ 70. (B) Age < 70. (C) White blood cell (WBC) count < 50 × 10^9^/L. (D) Bone marrow (BM) blast < 80%. (E) Lactic dehydrogenase (LDH) < 2 times upper normal limit (UNL). CAG = cytarabine, aclarubicin, and granulocyte colony-stimulating factor, IA = idarubicin plus cytarabine.

As shown in Fig. 3A and B, there was no significant difference in OS of age ≥ 70 years between IA and CAG group (*P* = 0.667), while those younger than 70 who received IA regimen seemed to have a better survival (*P* = 0.051). The long-term survival of IA-treated group was superior to CAG-treated group in patients with WBC count < 50 × 10^9^/L, LDH < 2 times UNL, or bone marrow blasts < 80% at diagnosis (Fig. 3C–E).

### 3.3 Risk of early death in induction

These 248 patients were divided into 2 groups, the early death group (died within 30 days after receiving induction chemotherapy) and the survival group (survived more than 30 days after receiving induction chemotherapy). The prognostic factors were compared between the 2 groups, and the result is shown in Table 5. Obviously, we found that patients in early death group had a higher proportion to carry adverse prognostic factors than patients in survival group.

**Table 5:**
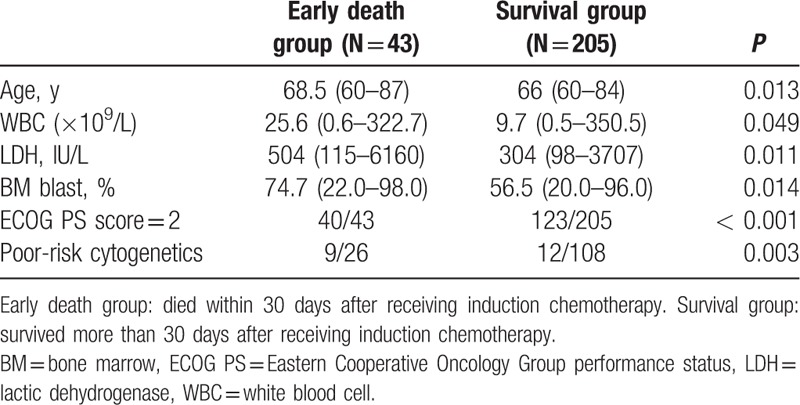
Comparison of prognostic factors between early death group and survival group.

### 3.4 Scoring model for prognosis prediction

**Figure 4:**
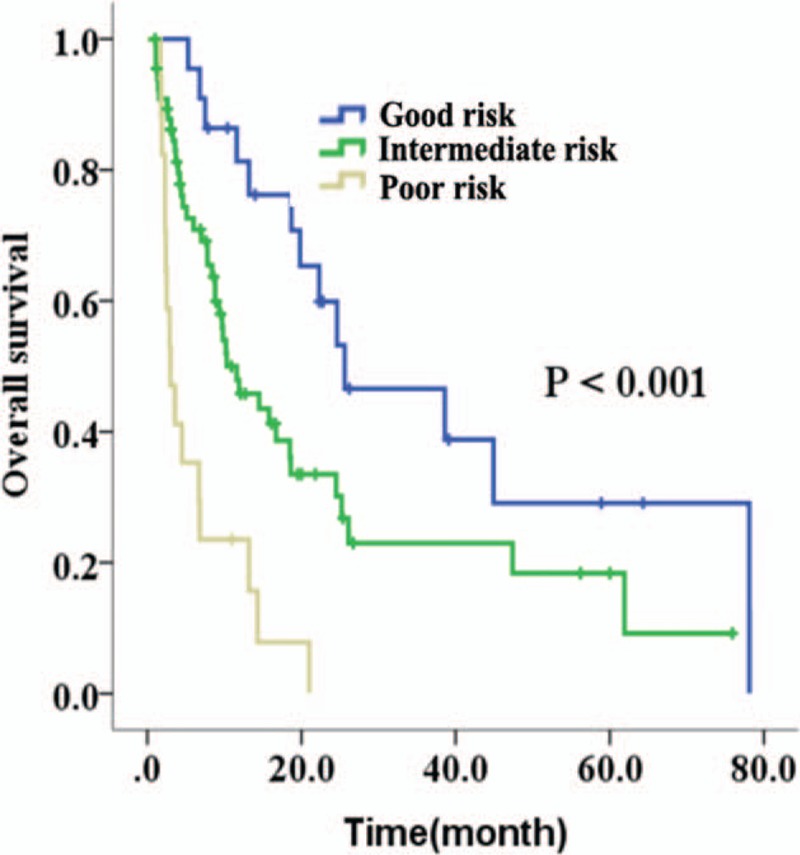
The overall survival (OS) of good-risk, intermediate-risk, or high-risk group based on the scoring model. Data from 106 cases were put into this scoring model.

Based on the results of univariate analysis, multivariate analysis, and some other studies, we constructed a simple scoring model for prediction of long-term survival (Table 6).^[16,17]^ A point of 1 was assigned to ECOG PS score ≤ 1 at diagnosis, WBC < 50 × 10^9^/L at diagnosis, LDH less than UNL at diagnosis, favorable-risk cytogenetics at diagnosis, or remission after induction. A point of 2 was assigned to age from 60 to 69 years at diagnosis, 1 to 2 times UNL of LDH at diagnosis, BM blasts of 20% to 79% at diagnosis, or intermediate-risk cytogenetics at diagnosis. A point of 3 was assigned to age ≥ 70 years at diagnosis, ECOG PS score = 2 at diagnosis, WBC ≥ 50 × 10^9^/L at diagnosis, more than 2 times UNL of LDH at diagnosis, BM blasts ≥ 80% at diagnosis, poor-risk cytogenetics at diagnosis, or nonremission after first induction cycle. According to this scoring model, we could stratify 3 risk groups, including good-risk group (9–12 points), intermediate-risk group (13–17 points), and poor-risk group (18–21 points), and the prognosis of good-risk group was significantly superior to intermediate-risk group and poor-risk group, as shown in Fig. 4 (*P* < 0.001). The median OS was 25.6 months in good-risk group, as compared to 10.3 months in intermediate group and 3.0 months in poor-risk group. Three-year and 5-year OS rates were 46.6% and 29.1% in good-risk group, whereas 23.0% and 18.4% in intermediate group, and 0.0% and 0.0% in poor-risk group, respectively.

**Table 6:**
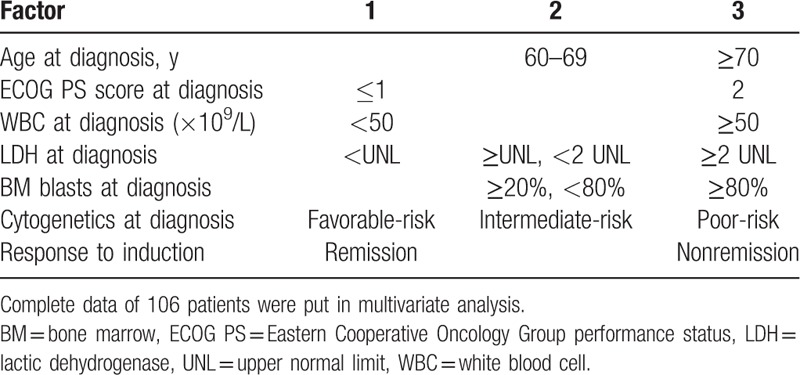
Scoring model for prognosis prediction.

## 4 Discussion

Acute myeloid leukemia is a disease of hematological malignancy, and the elderly account for most of this disease. Collaborative group studies and multicenter experiences have already increased the cure rate of AML patients during the last 3 decades. However, the majority of progress was made in young patients. During the past decades, only a slight improvement of CR rates and OS occurred for the elderly patients.^[18–20]^ According to numerous studies, at present, the CR rate after first induction cycle is about 40% to 60%, and the 5-year OS rate is approximately less than 20%.^[21–23]^ With reference to NCCN and ESMO guidelines, induction treatment of elderly patients includes standard-dose therapy, low-intensity therapy, and palliative care. However, the recommendation of induction regimen for elderly AML patients remains controversial.

In our study, patients’ baseline characteristics of IA and DA group had no significant difference. However, the long-term survival of DA regimen was significantly poorer than that of IA regimen, with 1-year OS rate of 39.8% and 3-year OS rate of only 8.3%. According to a study by Löwenberg et al,^[24]^ increasing the dose of daunorubicin to 90 mg/m^2^ resulted in a more rapid response and a higher response rate in elderly AML patients. However, a study by Burnett et al^[25]^ showed that there was no evidence that 90 mg/m^2^ was better than 60 mg/m^2^. Superior long-term outcome with idarubicin compared with high-dose daunorubicin at 80 mg/m^2^ was observed by Gardin^[26]^ in patients with AML aged 50 years and older. Increasing dose of daunorubicin might bring more benefit for elderly AML patients, but it still remained controversial. We were also not sure whether high dose of daunorubicin would be tolerated and beneficial for Chinese elderly patients. Further studies are still needed to explore the best dose of daunorubicin in DA regimen.

Since the outcome of IA-treated patients was significantly superior to that of DA-treated patients, we furthermore compared IA group with CAG group. We found that the WBC count, percentage of BM blasts, and LDH levels of the IA group were significantly higher than those of the CAG group, and all the above factors have been shown to be adverse prognostic factors for elderly patients with AML. Our results showed that patients treated with IA regimen for induction tended to have a higher CR rate and longer OS than those patients who received CAG regimens. It was worth noting that the long-term survival of IA group was significantly superior to CAG group in patients with WBC < 50 × 10^9^/L, LDH < 2 times UNL, BM blasts < 80%, or age < 70.

However, we should not ignore the fact that the median age of patients who received CAG regimen was significantly older than patients who received IA regimen, and the treatment outcome of AML appeared to be poor with increased age because of special biological characteristics.^[27]^ For most AML patients over age 70, intensive chemotherapy may not provide a benefit.^[28]^ In our study, no significant difference of OS was observed in patients older than 70 years between IA and CAG groups.

Thus, IA regimen might improve the CR rate and OS of elderly AML patients with good performance status compared with CAG regimens. Although the outcome of patients who received CAG regimen was poorer than IA regimen, CAG regimen still had therapeutic efficacy to some extent, with a 32.3% CR rate after first cycle and a 3-year and 5-year OS rate of 15.9% and 6.3%, respectively. It may be an alternative choice for those patients older than 70, or with poor ECOG PS.

Relapse of leukemia is common and challenging among the patients who had achieved CR.^[29]^ In our data, only one patient received Allo-HSCT for consolidation, and most of the other patients received high-dose cytarabine for consolidation. As a result, the relapse rate was 54.7% within 24 months, with the median time to relapse of 8.8 months, 13.7 months, and 11.5 months for the IA, DA, and CAG groups, respectively. Therefore, relapse is a major problem for the patients achieving remission. Some studies have already shown that some elderly patients would benefit if they receive Allo-HSCT for consolidation after first CR.^[30]^ However, the optimum time and method of transplantation needs to be further studied.

Our results were in accordance with some former studies, indicating that age, cytogenetic analysis, performance status, and WBC count played more important role in predicting survival outcome of elderly AML patients.^[31–33]^ The elderly AML patients were more likely to harbor poor-risk cytogenetics at diagnosis.^[34]^ Our data demonstrated that patients with poor-risk cytogenetics had statistically significant shorter OS in comparison to those with favorable or intermediate-risk cytogenetics. Meanwhile, we confirmed that poor ECOG PS is an adverse prognostic factor in this population of patients, as reported in other clinical studies.^[35]^ In NCCN guidelines, WBC more than 100 × 10^9^/L was considered as a negative prognostic factor in AML. However, such patients accounted for only a smaller portion of the elderly AML patients when being compared with younger patients. In our study, WBC counts higher than 100 × 10^9^/L were observed in only 13.7% of patients. We also found that patients with WBC counts at 50 to 100 × 10^9^/L or more than 100 × 10^9^/L had no significant difference in OS, but both of them were poorer than those with WBC counts less than 50 × 10^9^/L. Thus, we set the cut-off at 50 × 10^9^/L of WBC count, as it seemed more suitable for elderly AML. The cut-off percentage of BM blasts was similar to WBC cut-off count. We found that the OS of BM blast 20% to 49% group was not significantly different from the 50% to 79% group, but both groups had better OS than the over 80% group. As a result, we set the cut-off blast percentage at 80%.

We noticed that the response to the first induction cycle, percentage of BM blasts, and elevated LDH were also prognostic factors in elderly AML patients. It was noteworthy that only nonremission after first induction cycle and LDH ≥ 2 times UNL were significantly independent prognostic parameters of OS, and nonremission after first induction cycle was the most significant of all these adverse prognostic factors.

As shown in Table 5, we found that patients in early death group had a higher proportion to carry adverse prognostic factors than patients in survival group. Therefore, taking all the above 6 adverse prognostic factors into consideration, we could identify the patients who might be at high risk for inferior consequences upon receiving chemotherapy. We analyzed the cause of early death for each patient. We found that 79.1% of patients died of uncontrolled infections, frequently pulmonary infection. Because the hematopoietic function of bone marrow was difficult to recover after chemotherapy, the infection was hardly to be controlled. Therefore, we should consider palliative treatment or the best supportive treatment for these patients with many adverse prognostic factors who might not benefit from standard-dose or low-intensity chemotherapy.

Some former studies built a few prediction systems of OS by scoring the prognostic factors in some methods.^[36,37]^ Our result of univariate analysis showed that the following factors, such as age, ECOG PS, cytogenetics, WBC counts, percentage of BM blast, LDH at diagnosis, and the response to induction therapy were important prognostic factors. Thus, taking these 7 factors into consideration, we constructed a scoring model for prediction of prognosis. As shown in Table 6 and Fig. 4, it is a simple and valid scoring system, which could stratify the good-risk, intermediate-risk or poor-risk patients, and could be easily used in daily clinical practice. It was worthy to be noticed that patients in good-risk group could have a good prognosis even if they received only chemotherapy, with 3-year and 5-year OS rates at 46.6% and 29.1%, respectively. However, this scoring model could not help us to decide which regimen should be used, and there are still no reported scoring systems that would help us to make this decision by now.

There were still some limitations that must be considered when interpreting these results. Because of retrospective design and long period of observation, some information bias could not be avoided. Patients’ final selection of IA, DA, or CAG regimen was partly affected by doctors’ suggestion, which led to different patient baseline characteristics of 3 groups. Recently, some novel agents, such as methyltransferase inhibitors, are increasingly used in elderly AML, but they are still not the front-line induction agents in China. We did not compared intensive chemotherapy with DNA methyltransferase inhibitors in this study, but we are planning a prospective clinical trial in order to prove which one is a better strategy for elderly patients with AML. Thus, a multicenter prospective clinical study is needed to evaluate which regimen and dosage are more beneficial for elderly patients with AML.

In conclusion, the prognosis of elderly patients with AML remained poor. Standard-dose IA regimen could improve the CR rate and prolong the survival time compared with standard-dose DA or low-intensity CAG regimen in elderly AML patients with good performance status. Relapse was still a serious problem for those who only received high-dose of cytarabine for consolidation after CR. Lactic dehydrogenase more than 2 times UNL at diagnosis and nonremission after first induction cycle were most significantly adverse prognostic factors of OS. All prognostic factors should be considered before induction therapy in order to assure that each patient receives the best, individualized treatment plan.

## Acknowledgments

The authors thank all who helped them during this research.
